# Tumeur blanche du coude: une atteinte tuberculeuse synoviale rare, la tuberculose aux grains riziformes

**DOI:** 10.11604/pamj.2013.15.150.1353

**Published:** 2013-08-27

**Authors:** Amen Dhaoui, Issam M'sakni

**Affiliations:** 1 Service d'Anatomie et de Cytologie Pathologiques de l'Hôpital Militaire de Tunisie; 2 Hôpital Militaire Principal d'Instruction de Tunis, Montfleury Tunis 1008, Tunisie

**Keywords:** Tumeur blanche, tuberculose, white tumor, tuberculosis

## Image en médecine

La synovite tuberculeuse aux grains riziformes du coude: Aspect macroscopique d'une tumeur blanche kystisée. Son contenu est fait par les grains riziformes baignant dans le liquide synovial.

**Figure 1:**
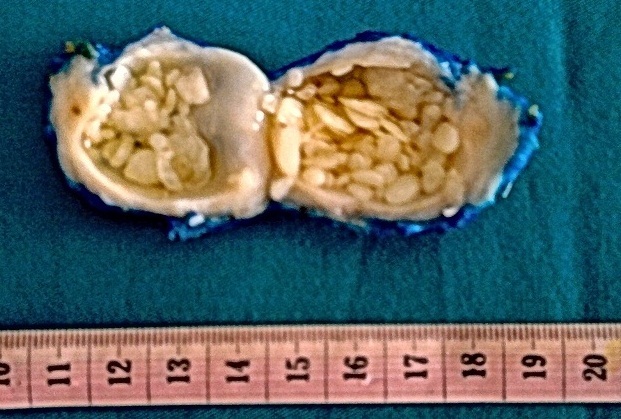
La synovite tuberculeuse à grains riziformes du coude: Aspect macroscopique d'une tumeur blanche kystisée. Son contenu est fait par les grains riziformes baignant dans le liquide synovial

